# Does Oral Implant Design Affect Marginal Bone Loss? Results of a Parallel-Group Randomized Controlled Equivalence Trial

**DOI:** 10.1155/2018/8436437

**Published:** 2018-01-31

**Authors:** Benedikt C. Spies, Maria Bateli, Ghada Ben Rahal, Marin Christmann, Kirstin Vach, Ralf-Joachim Kohal

**Affiliations:** ^1^Department of Prosthetic Dentistry, Center for Dental Medicine, University Medical Center Freiburg, Faculty of Medicine, University of Freiburg, Freiburg, Germany; ^2^Department of Prosthodontics, Geriatric Dentistry and Craniomandibular Disorders, Charité Center for Dental and Craniofacial Sciences (CC3), Charité-Universitätsmedizin Berlin, Campus Benjamin Franklin (CBF), Berlin, Germany; ^3^Private Practice, Freiburg, Germany; ^4^University Medical Center Freiburg, Center for Medical Biometry and Medical Informatics, Institute for Medical Biometry and Statistics, Freiburg, Germany

## Abstract

**Objective:**

To test whether or not the modified design of the test implant (intended to increase primary stability) has an equivalent effect on MBL compared to the control.

**Methods:**

Forty patients were randomly assigned to receive test or control implants to be installed in identically dimensioned bony beds. Implants were radiographically monitored at installation, at prosthetic delivery, and after one year. Treatments were considered equivalent if the 90% confidence interval (CI) for the mean difference (MD) in MBL was in between −0.25 and 0.25 mm. Additionally, several soft tissue parameters and patient-reported outcome measures (PROMs) were evaluated. Linear mixed models were fitted for each patient to assess time effects on response variables.

**Results:**

Thirty-three patients (21 males, 12 females; 58.2 ± 15.2 years old) with 81 implants (47 test, 34 control) were available for analysis after a mean observation period of 13.9 ± 4.5 months (3 dropouts, 3 missed appointments, and 1 missing file). The adjusted MD in MBL after one year was −0.13 mm (90% CI: −0.46–0.19; test group: −0.49; control group: −0.36; *p* = 0.507).

**Conclusion:**

Both implant systems can be considered successful after one year of observation. Concerning MBL in the presented setup, equivalence of the treatments cannot be concluded.

**Registration:**

This trial is registered with the German Clinical Trials Register (ID: DRKS00007877).

## 1. Introduction

Primary stability represents the most effective surgical strategy to protect an implant from micromovements during the healing period, considered a prerequisite for successful osseointegration [1]. Retention is mainly influenced by the implant design on the macro and micro level and the platform/core diameter in relation to the underprepared dimension of the bony bed. After installation, peri-implant osseous remodeling occurs. This process, finally resulting in “secondary stability,” was shown to be most dominant at the implant neck area [2].

A significantly different susceptibility for both early and late implant loss was found for different market-available implant brands, besides surface modifications potentially owed to the implant design in relation to the drilling protocol [3]. Various modifications of the implant design have been proposed to increase primary stability and to minimize or prevent marginal bone loss, mostly addressing the implant neck area [4]. One of the most evaluated design options represents the use of platforms/abutments with a reduced diameter [5], resulting in less MBL at implants with the so-called “platform switching” than at implants with matched platforms [6, 7]. Another popular design modification is represented by the usage of microthreads at the implant neck area [8], assumed to enhance the bone-to-implant contact [9]. Furthermore, this design modification was found to be more effective in reducing shear stress in the crestal area, demonstrating the biomechanical rationale in reducing the risks of marginal bone loss caused by overloading [10, 11]. However, clinical relevance is a topic of controversial discussion [12] and a lack of randomized controlled clinical trials evaluating the outcome of such systems in direct comparison with standard designs was claimed [13].

To date, there is no sufficient clinical data available in the literature on whether and to what extent an underpreparation of the osteotomy in relation to the core diameter of the implant (in order to increase primary stability) is advantageous in preserving the marginal bone level [14, 15]. The SICmax implant (SIC invent AG, Basel, Switzerland) is, among others, characterized by a microthreaded conical segment in the crestal area and an increased diameter (0.2 mm) compared to the conventionally designed SICace implant. Despite an increased diameter, it needs to be installed in a bony bed prepared by the same drilling sequence, therefore consisting of identical dimensions. This aims to increase primary stability in soft bone.

The primary aim of this clinical investigation was to compare the radiographically determined marginal bone loss of both implant systems one year after installation. Equivalence by means of marginal bone level stability with the well-documented SICace control implant [16] should be shown. The secondary objective was to evaluate the clinical and patient-reported outcome, measured by means of several soft tissue parameters and treatment-related questionnaires.

## 2. Materials and Methods

### 2.1. Study Design

The present investigation was designed as a randomized controlled equivalence trial with a two-group design. It was registered in the German Clinical Trials Register (ID: DRKS00007877) and is listed in the WHO International Clinical Trials Registry Platform. The proposed null hypothesis was that there would be a difference in marginal bone loss (MBL) between the two implant designs. Following approval from the ethics committee of the university's medical center (investigation number: 174/12, 05/02/2012), partially edentulous patients requesting fixed rehabilitation were asked to comply with the prescribed treatment protocol and to sign informed consent. This randomized equivalence trial was performed in accordance with the Declaration of Helsinki of 1975, as revised in 2013, and reported according to the CONSORT statement [17].

### 2.2. Subject Population

Study patients were recruited from those attending a fixed treatment from the faculty members of the Department of Prosthetic Dentistry (University Medical Center Freiburg, Germany). An initial screening included a clinical examination to evaluate for sufficient inclusion criteria, a CBCT to guarantee sufficient bone volume in the area of interest allowing surgery without augmentation procedures, and impression taking for the fabrication of casts to be digitized and matched with the CBCT data. This allowed for a three-dimensional planning of the implant position and the fabrication of surgical guides. Inclusion criteria for the study patients were good general health and absence of infectious disease, diabetes, osteopathy, and radiation therapy in the head and neck area. Other requirements included the ability to give informed consent to participate, good oral hygiene, no active periodontitis, no drugs influencing bone metabolism, no smoking, and no pregnancy.

### 2.3. Randomization and Allocation Concealment

Consecutive patients fulfilling the inclusion criteria were randomized to receive either test or control implants based on a randomization list generated with nQuery Advisor 6.0 using random block sizes (Statistical Solutions, Saugus, MA, USA). This list was generated by a statistician not involved in the treatment. Allocation concealment was performed with sequentially numbered, opaque, sealed envelopes. Patients were assigned in a chronological order of their admission to the department. The envelopes were opened sequentially by a study nurse after the participant's name and other details were written on the appropriate envelope. The treatment sequence was concealed until interventions were assigned. In those cases where patients required more than one implant (i.e., in case of a FDP or multiple SCs/FDPs), all implants to be placed in this patient were either test or control implants and were included in the analyses. Neither patients nor dentists administering the interventions and assessing the outcomes were blinded to group assignment.

### 2.4. Study Implants

**Figure 1:**
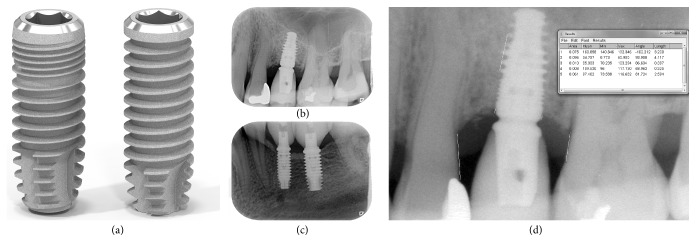
Representative test ((a), left: SICmax, SIC invent AG, Basel, Switzerland) and control implant ((a), right: SICace) of comparable dimensions (platform: 4.2/4 mm, length: 11.5 mm) to be installed in identical implant bed dimensions and exemplary standardized radiographs of a test implant installed in the maxilla (b) and two adjacent control implants installed in the mandible (c) at the 1-year follow-up. (c) shows representative measurements of MBL at the mesial and distal aspect of a test implant with the image analysis software (ImageJ) to be compared with the baseline values gathered at implant installation.

The design of both the test (SICmax) and the control (SICace) implants consisted of an internal hexagonal connection and a roughened (Ra: 0.75), sand blasted, and acid-etched surface (Figure 1(a)). Both implant types were designed with a beveled (45°) platform switching shoulder and a self-tapping thread design. The amount of platform switch (0.1–0.4 mm), the size of the bevel, and the pitch distance at the implant body varied depending on the implant platform but to the same platform-related extent in both implant systems. Despite an identical platform-related pitch distance, test implants had a sharper flank and thread angle, resulting in an increased width of the thread groove. The control implant had a progressive apical cutting edge, whereas the test implant had a rounded apical base. When compared to the control implants, the upper part of the test implants revealed a conically increasing core diameter (minor diameter) and was equipped with microthreads in this region. Moreover, test implants showed an increased outer diameter (major diameter: +0.2 mm) compared to the corresponding platform of the control implants to be installed in identical implant bed dimensions. The design of the test implants was driven by the intention to achieve higher primary stability in soft bone. However, in the present evaluation, both implant types were placed according to the above-mentioned randomization procedure regardless of the bone quality. All products used (surgical and prosthetic) were registered and commercially available products. Dimensions of test and control implants can be found in Table 1.

### 2.5. Surgical Procedures

Mucoperiosteal flaps were raised by means of crestal incisions and implants were inserted at the bone level (i.e., all areas of the implant flush with the crestal bone). Drilling was performed according to the manufacturer's recommendation. Implant position was guided according to the three-dimensional planning (before 04/2014: implant3D, med3D, Heidelberg, Germany; after 04/2014: SMOP, Swissmeda, Zurich, Switzerland). Primary stability was assessed by direct torque wrench testing. Implants were left for submerged healing. In order to monitor the marginal bone loss at implant sites, an individualized intraoral X-ray film holder was constructed after implant placement to facilitate the acquisition of standardized radiographs. Patients were provided with home-care maintenance instructions and scheduled for postoperative checkups on an individual basis. After 7 to 10 days, sutures were removed. In case of multiple-unit FDPs, removable interim prostheses were not incorporated within the first 14 days after surgery.

### 2.6. Restorative Procedures

In both groups, impressions were taken three months after surgery at the implant level with the open tray impression technique. Reopening was performed 2 weeks before impression taking. Final prostheses were delivered 4–6 months after implant placement. All restorations were screw-retained. Digital standardized periapical radiographs and clinical measurements were taken after definitive prosthesis installation.

### 2.7. Clinical Assessments

Follow-ups were scheduled one year after implant installation. The follow-ups involved the soft tissue parameters probing depth (PD), clinical attachment level (CAL), gingival recession (GR), modified bleeding index (mBI), and modified plaque index (mPI), the latter two according to Mombelli et al. [19]. The parameters PD, CAL, and GR were measured to the nearest millimeter with a periodontal probe. Values gathered at prosthetic delivery were used for comparison. Occlusal/incisal reference points defined at baseline served as landmarks for the measurements.

### 2.8. Radiographic Assessment

After implant placement, standardized radiographs were taken using a customized film holder. Standardization of the radiographs was achieved using individual silicon bite registrations. Further radiographs were taken after final crown/bridge insertion and at the one-year follow-up (Figures 1(b) and 1(c)). Implant installation served as the baseline for the marginal bone level. Changes in interproximal bone levels were recorded by measuring the distance from the implant shoulder to the first visible bone-to-implant contact at the mesial and distal aspect of each implant with image analysis software (ImageJ, National Institutes of Health, Bethesda, MD, USA; Figure 1(d)). Magnification correlation was achieved by calibrating all images using the known pitch distance.

### 2.9. Patient-Reported Outcome Measures (PROMs)

The patients' appraisals of function, esthetics, sense, speech, and self-esteem have been assessed at the pretreatment examination, at the delivery of the final prosthetic restoration, and at the follow-up sessions applying visual analogue scales (VAS). To permit a standardized procedure, the patients were asked to mark on a line (10 cm, no scale) the point that corresponds most with their subjective perception. The left end point represented “poor satisfaction” (0%) and the right one “excellent satisfaction” (100%). Patients' markings were measured with a ruler (1 mm corresponds to 1%).

### 2.10. Survival/Success Criteria

Criteria of van Steenberghe [20] were applied. A “successful implant” was defined as an implant which does not cause allergic, toxic, or gross infectious reactions either locally or systemically, offers anchorage to a functional prosthesis, does not show any signs of fracture or bending, does not show any mobility, and does not show any signs of radiolucency on an intraoral radiograph. A “surviving implant” was defined as an implant which remains in the jaw and is stable, while the subject's treatment is functionally successful even though all the individual success criteria are not fulfilled.

### 2.11. Statistical Analyses

The primary outcome of this study was to evaluate the hypothesis of equivalence of the two treatment groups in terms of radiographically determined marginal bone loss (MBL) [mm] on the mesiodistal aspects of the implants. At study outset, it was decided that equivalence would be declared if the two-sided (1 − 2*α*)*∗*100% confidence interval (CI) for the difference in means (Δ_*E*_: *μ*_1_ − *μ*_2_) was found to be within the equivalence margins of *ε*_1_/*ε*_2_ = 0.25 mm. A total of 34 patients with one implant each per study arm were deemed necessary to demonstrate equivalence between treatment groups with 80% power at *α* = 0.05. The assumed common standard deviation in each arm of *σ*^2^ = 0.35 mm was derived from a recent study evaluating the control implant [16]. The 90% CI for *μ*_1_ − *μ*_2_ was constructed by fitting linear mixed models with random intercepts for each patient analyzing the difference in MBL in both groups from implant installation to the one-year follow-up. The calculated sample size of 34 patients per group was not reached within a sufficient time period but, according to the study protocol, placement of multiple implants per subject was allowed and performed (2.4 implants per patient on average). Therefore, estimation of the intraclass correlation coefficient (ICC) for MBL was performed once it was concluded that recruitment of the patient sample size had failed. The ICC was calculated after one year of observation and found to be 0.08. Therefore, a total of 34 implants (15 subjects) in the control group and 47 implants (18 subjects) in the test group, as available for the one-year measurements, were deemed sufficient for statistical analyses. Data on the true difference (Δ_*E*_) between the treatments was analyzed to reject the null hypothesis (*H*_0_: Δ_*E*_ > *ε*_1_ or Δ_*E*_ < −*ε*_2_), favoring the alternative hypothesis (*H*_*a*_: −*ε*_2_ ≤ Δ_*E*_ ≤ *ε*_1_).

Furthermore, linear mixed models with random intercepts were fitted for each patient to assess time, group, and time-group interaction effects on the response variables of the radiographic and clinical assessments (MBL, PD, CAL, GR, mBI, and mPI).

For the PROMs, a paired *t*-test was used to calculate the changes between the surveys before the treatment and after prosthetic delivery. The method of “Bonferroni” was applied to correct for the multiple testing problem. Linear mixed models with random intercepts were fitted for each patient to evaluate time effects on patient satisfaction after prosthetic delivery.

The calculations were performed with the statistical software STATA 14.2 (StataCorp LT, College Station, TX, USA) using “xtmixed.” The probability level for statistical significance was set to *p* < 0.05.

## 3. Results

### 3.1. Status of Follow-Up

A total of 40 patients were recruited and randomized (04/2011–12/2014, Figure 2). Implant surgery took place until 03/2015. Thirty-three patients were available for follow-up after a mean observation period of 13.9 ± 4.5 months (range: 11–32 months; 11/2013–03/2016). Two patients refused further participation, one file was missing, one patient dropped out due to financial reasons (no prosthetic delivery), and three patients missed appointments. 81 implants (47 test, 34 control; Table 1) were evaluated and analyzed (“per protocol analysis”). This resulted in 2.6 (range: 1–7) and 2.3 (range: 1–4) implants per patient (range: 1–7) in the test and control group, respectively. Implants were restored with 50 SCs and 15 FDPs (test group: 8 three-unit bridges; control group: 6 three-unit bridges, 1 five-unit bridge). Of these restorations, 4 were opposed by implant-supported removable dental prostheses (equally distributed to both groups) and 61 by fixed restorations supported by either teeth or implants. The mean age at the timepoint of implant installation was 58.2 ± 15.1 years.

### 3.2. Life Table Analysis

No implant failures or adverse events were observed. Survival and success rates were 100% in both groups after one year of observation.

### 3.3. Radiographic Outcome (Figure 3, Table 2)

Overall, an average bone loss of 0.44 mm was observed from implant insertion to the one-year follow-up (*p* < 0.001; test group: 0.50 ± 0.7 mm, control group: 0.35 ± 0.9 mm). No significant difference was detected between groups (*p* = 0.507). After the delivery of the final restorations, the bone levels showed no statistically significant changes over time in both groups (*p* ≥ 0.385) and, furthermore, no difference between groups (*p* = 0.256). However, the adjusted difference in mean MBL after one year was −0.13 mm (90% CI: −0.46–0.19 mm; test group: −0.49 mm, control group: −0.36 mm). According to the defined equivalence margins at study outset (0.25 mm), no equivalence of the treatments could be concluded.

Type of restoration (SC/FDP; *p* = 0.761), sex (*p* = 0.672), location (anterior/posterior; *p* = 0.533), jaw (*p* = 0.833), implant platform (3/4/5 mm; *p* = 0.249), implant length (7.5–14.5 mm; *p* = 0.421), bone quality (1–4 according to Lekholm and Zarb [18]; *p* = 0.428), bone quantity (A–E according to Lekholm and Zarb [18]; *p* = 0.437), grafting procedure (GBR/no grafting; *p* = 0.409), insertion torque (*p* = 0.684), and bone anchorage (mono/bicortical; *p* = 0.496) had no significant influence on the marginal bone level changes over time.

### 3.4. Clinical Outcome (Figure 3, Table 2)

Throughout the groups, values of the modified bleeding index increased significantly (*p* < 0.001), whereas a coronal rebound of the gingival margin following prosthetic delivery was observed (*p* = 0.001). A significant difference between the groups when comparing the values at the one-year follow-up with the ones at prosthetic delivery was only found for the modified bleeding index in favor of the test implants (*p* = 0.025). Evaluating both groups separately, control implants showed significantly increased probing depth (*p* = 0.001) and plaque scores (*p* = 0.001) after one year, whereas the clinical attachment level decreased significantly (*p* = 0.013) and a coronal rebound of the gingival margin (*p* < 0.001) was observed around the test implants.

### 3.5. Patient-Reported Outcome Measures (PROMs; Figure 4, Table 3)

Compared to the pretreatment situation (46.8–68.2%), all assessments revealed significantly improved average VAS values at the delivery of the prosthetic restorations (77.6–89.5%; *p* ≤ 0.0007). This significant improvement remained stable in all assessed categories until the one-year follow-up (83.4–92.7%; *p* ≥ 0.130). No differences regarding the implant type could be detected (*p* ≥ 0.160).

## 4. Discussion

In the field of implant dentistry, clinical trials whose purpose is to show equivalence of two or more treatments are traditionally using methods for demonstrating superiority, and if no statistical differences are found, the treatments are erroneously claimed to be equivalent [21]. However, equivalence can only be claimed when both upper and lower bounds of the CI for the mean difference are included in an a priori specified equivalence range based on assumed or proven clinical relevance [22]. The two-sided confidence level of the interval can be determined with the formula 1 − 2*α∗*100%. Since a one-sided 5% significance level was deemed acceptable for the hypothesis test of the present investigation, a 90% two-sided CI could then be used [17]. Nevertheless, the equivalence test based upon it has a significance level of *α* rather than 2*α* [23]. The rationale for equivalence testing in the present trial was to compare the MBL of the modified design of the test implant (intended to increase primary stability in soft bone) with the well-documented and successful outcome of the control implant. The statistical analyses of the present work revealed an adjusted difference in mean MBL after one year of −0.13 mm and a 90% CI ranging from −0.46 mm to 0.19 mm. According to the defined equivalence margins at study outset, no equivalence of the treatments could be concluded. This was mainly attributed to the high standard deviations of bone level measurements in both the test (0.7 mm) and the control (0.9 mm) groups as when compared to the values used as a reference (0.35 mm) at the timepoint of study protocol proposal [16]. This might be due to extended inclusion criteria (e.g., implant installation in both the maxilla and the mandible, restoration with both SCs and FDPs) applied in the present investigation.

The test implants of the present investigation did not solely differ from the control implants in the neck area. However, besides an increased diameter, implant neck configuration might be considered the most relevant difference between both systems and the only design aspect where comparable literature is available. For this purpose, the work from one group appears the most suitable for comparison [24–27]. The majority of other nowadays available studies are either missing a control group or do not solely focus on modifications of the implant macrodesign, waiving the inclusion of confounding covariables to be investigated [13]. Referring to the investigations of Moon and his coworkers, all studies included two groups of implants of the same brand, dimensions, surface, and implant-abutment connection to be radiographically monitored with a follow-up time ranging from one to three years. Two of these publications purely addressed the presence of microthreads in the crestal part of the implants (test group) in comparison with control implants comprising a macrothreaded neck [24, 25], whereas the other two trials included implants with microthreads in both groups and focused on the axial configuration of the implant neck (conical versus straight) [26] or the exact location of the threads (at the top of the fixture versus 0.5 mm below the top of the fixture) [27]. In the following, the two first-mentioned publications [24, 25] will be compared with the results of this work. In one trial, 17 patients were recruited and two types of implants were installed. Both implants were installed adjacent to each other within the same subject and equally distributed to the jaws within the whole population. Finally, MBL was evaluated radiographically. As a result, the mean MBL of both implants differed significantly in favor of the microthreaded implant after three years of observation (0.24 mm with microthreads, 0.51 mm without microthreads, *p* = 0.001). Despite no significant difference found in the present work, the calculated mean MBL of the SIC implants can be considered to be within the same range (0.35/0.50 mm). No soft tissue evaluations were performed in the study of Lee and colleagues [25]. In the second trial, two groups of implants were placed next to each other in partially edentulous areas of 20 patients. The difference in mean MBL after one year of observation (0.15 mm versus 0.13 mm) was found to be not statistically significant (*p* = 0.669), which is in accordance with the present findings. However, compared to our findings, these values appear to be smaller. In addition, peri-implant soft tissue response was evaluated and the average plaque index (mPI; 0.35 and 0.4) and bleeding index (mBI; 0.28 and 0.21) revealed no significant group difference. Compared to the mBI and mPI values of this work, both indices are in the same range. Other soft tissue variables like probing depth, clinical attachment level, and gingival recession were not addressed. When focusing on these variables, the test implant of this study showed a slightly superior outcome. In contrast to the control, the test implants gained attachment (*p* = 0.013) and showed a coronal rebound of the gingival margin following prosthetic delivery (*p* < 0.001), whereas increased values for probing depth were found at the control implants (*p* = 0.001). Besides a potential influence of the implant design, oral hygiene of the study participants might have influenced these findings. In contrast to the test group, plaque index increased at the control implants (*p* = 0.001). Moreover, bleeding was found to increase in both groups with a higher incidence at the control implants (*p* = 0.025).

The modified design of the test implant aims to increase primary stability. However, only a slightly increased insertion torque was found for the test group in the present investigation compared to the control (Table 1). This might be explained with an uneven distribution of implant sites between both groups despite randomization. Considering the distribution of implant sites between both groups, one could conclude that the design of the test implant resulted in a slightly increased insertion torque despite being inserted more often in the maxilla and consecutively softer bone conditions.

In terms of the study design, participants were randomly assigned to either the test or the control implant group. This represents a limitation of the present investigation, since a more conclusive study design would have included patients requiring bilateral implant placement (right and left) or patients requiring two dental implants that need to be placed next to each other. In those case scenarios, one can control patient variables as much as possible. Implants placed in the same individual are subject to similar healing and possibly similar bone resorption phenomena and it is possible that placing only one type of implant in each subject might enhance the problem because it could enhance either a positive or a negative outcome for a given subject. However, the ICC for MBL was calculated after one year of observation and found to be 0.08 as mentioned above.

Radiographic and clinical parameters can only provide an objective, however, limited understanding of oral health outcomes in dental implant therapy. Therefore, it is strongly recommended to consider PROMs as well. Across the groups, an increased satisfaction of the participants could be observed immediately after the treatment (i.e., prosthetic delivery). The surveys at the one-year follow-up showed no reduction of the positive effect, revealing continuously improved VAS values. No group differences were found. Thus, from the patients' point of view, both presented treatments proved to address their needs with a lasting positive effect.

## 5. Conclusions

According to the defined limits of equivalence, the test implant failed to reach equivalence in maintenance of MBL. The null hypothesis could not be rejected. However, considering the survival/success rates and the average bone loss of 0.50/0.35 mm after one year, both implant systems show promising results and can be recommended for clinical usage. Furthermore, both systems highly satisfied patients' needs.

## Figures and Tables

**Figure 2:**
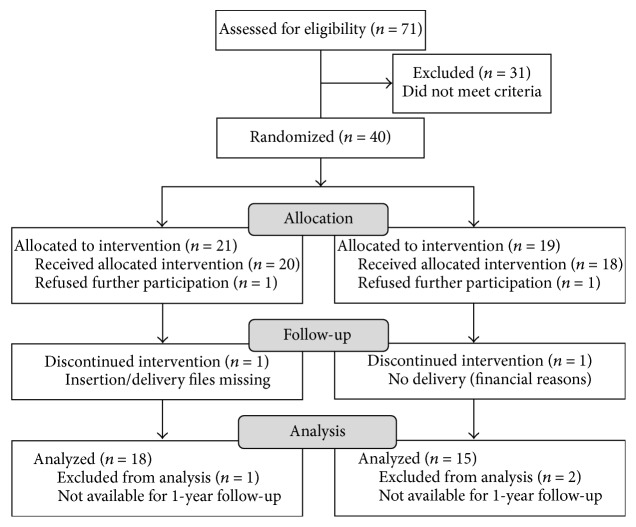
CONSORT flow diagram (Consolidated Standards of Reporting Trials, accessed at http://www.consort-statement.org/consort-statement/flow-diagram) showing the flow of participants through the trial.

**Figure 3:**
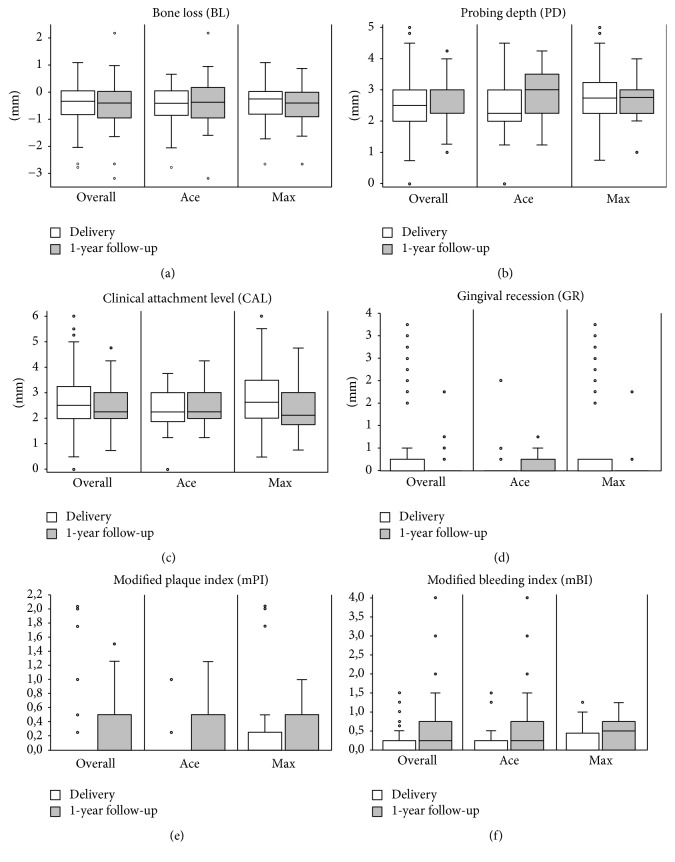
Box plot diagrams of the radiographic outcome ((a) marginal bone loss) and clinical outcome ((b) probing depth, (c) clinical attachment level, (d) gingival recession, (e) modified plaque index, and (f) modified bleeding index) at prosthetic delivery and 1-year follow-up.

**Figure 4:**
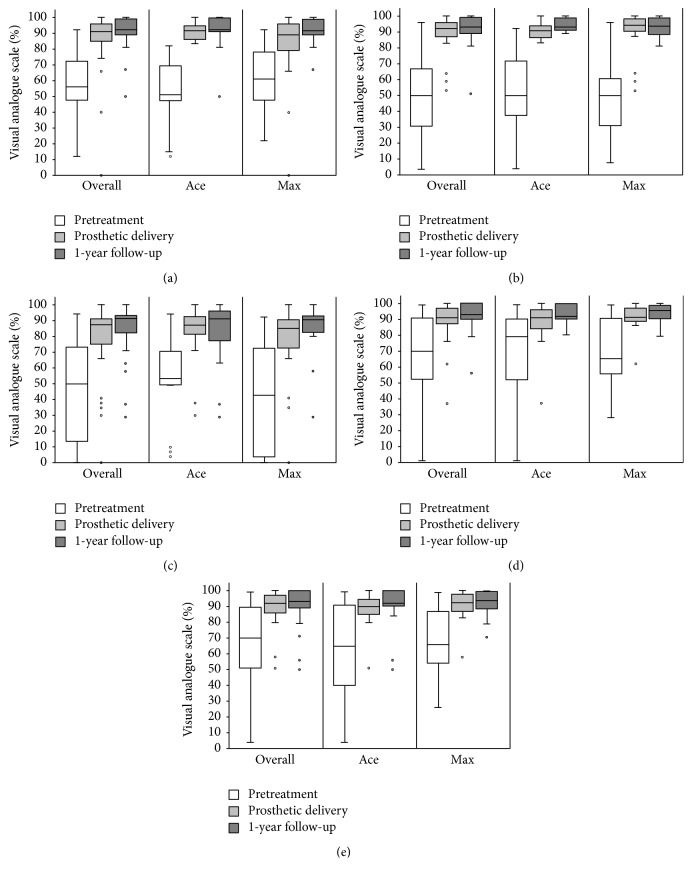
Box plot diagrams of patient-reported outcome measures (visual analogue scales [%]; (a) function/eating, (b) esthetic/appearance, (c) sense, (d) speech, and (e) self-esteem) prior to treatment, at prosthetic delivery, and at the 1-year follow-up.

**Table 1:** Distribution and baseline data of test and control implants.

	Test group (SICmax)	Control group (SICace)
Patients	15	18
Sex (females/males)	6/9	7/11
Age (years)	62.3 ± 12.6^a^	52.6 ± 16.8^a^
Implants	47	34
Jaw (maxilla/mandible)	35/12	17/17
Location (anterior/posterior)	2/45	3/31
Platform^b^ (3.7/3.4/4.2/4.0/5.2/5.0) (mm)	8/29/10	7/22/5
Length (7.5/9.5/11.5/13/14.5) (mm)	3/18/16/10/-	5/9/11/8/1
Bone quality^c^ (1/2/3/4)	6/21/15/5	1/9/20/4
Insertion torque (Ncm)	36.5 ± 9.9^a^	33.9 ± 10.8^a^
Restorations	37	28
Type (SCs^d^/FDPs^e^)	29/8	21/7

^a^MV ± SD. ^b^test/control. ^c^According to Lekholm and Zarb [18]. ^d^Single crowns. ^e^Fixed dental prostheses.

**Table 2:** Radiographic and clinical outcomes.

		Prosthetic delivery (D)			1-year follow-up (1 y)			Significance (p)^a^	
		*n*	Mean	SD	*n*	Mean	SD	D → 1 y	Ace/Max
*Bone level (BL)*									0.256
Overall	[mm]	*81*	−**0.43**	0.7	*80* ^c^	−**0.44**	0.8	0.984	
Ace	[mm]	*34*	−**0.49**	0.7	*33*	−**0.35**	0.9	0.386	
Max	[mm]	*47*	−**0.39**	0.7	*47*	−**0.50**	0.7	0.385	
Significance (p)^b^			0.558			0.507			
*Probing depth (PD)*									0.424
Overall	[mm]	*79*	**2.6**	1.0	*81*	**2.7**	0.8	0.223	
Ace	[mm]	*32* ^d^	**2.4**	0.9	*34*	**2.9**	0.8	**0.001**	
Max	[mm]	*47*	**2.7**	1.0	*47*	**2.6**	0.7	0.628	
Significance (p)^b^			0.504			0.447			
*Clinical attachment level (CAL)*									0.063
Overall	[mm]	*79*	**2.6**	1.3	*81*	**2.4**	0.8	0.303	
Ace	[mm]	*32* ^d^	**2.3**	1.2	*34*	**2.5**	0.8	0.113	
Max	[mm]	*47*	**2.8**	1.4	*47*	**2.4**	0.9	**0.013**	
Significance (p)^b^			0.605			0.213			
*Gingival recession (GR)*									0.440
Overall	[mm]	*79*	**0.4**	0.9	*81*	**0.1**	0.3	**0.001**	
Ace	[mm]	*32* ^d^	**0.2**	0.5	*34*	**0.2**	0.3	0.633	
Max	[mm]	*47*	**0.6**	1.1	*47*	**0.1**	0.4	**<0.001**	
Significance (p)^b^			0.507			0.695			
*Modified plaque index (mPI)*									0.116
Overall		*79*	**0.3**	0.6	*81*	**0.3**	0.4	0.889	
Ace		*32* ^d^	**0.1**	0.3	*34*	**0.3**	0.5	**0.001**	
Max		*47*	**0.4**	0.7	*47*	**0.2**	0.3	0.072	
Significance (p)^b^			0.584			0.171			
*Modified bleeding index (mBI)*									**0.025**
Overall		*79*	**0.2**	0.4	*81*	**0.5**	0.6	**<0.001**	
Ace		*32* ^d^	**0.2**	0.4	*34*	**0.6**	0.9	**0.005**	
Max		*47*	**0.2**	0.3	*47*	**0.4**	0.4	**0.006**	
Significance (p)^b^			0.468			**0.033**			

^a^Linear mixed models: changes between D and 1 y and differences regarding the implant type (Ace/Max). ^b^Linear mixed models: for every timepoint (D, 1 y) regarding the implant type (Ace/Max). ^c^One radiograph (one control implant) at 1 y was insufficient for BL measurements. ^d^One file (two control implants) including clinical measurements was missing for D.

**Table 3:** Patient's assessment of function (eating), esthetic and appearance, sense (“feeling like my own teeth”), speech, and self-esteem (visual analogue scale, [%]) before treatment (P), at prosthetic delivery (D), and at the 1-year follow-up (1 y).

	P	D	1 y	Significance (p)		
				P → D^a^	D → 1 y^b^	Ace/Max^c^
*Function (eating)*						
* n*	*33*	*32* ^d^	*33*			
Mean [%]	**56.5**	**85.5**	**91.3**	<0.0001	0.130	0.254
SD	21.5	19.6	10.5			
*Esthetic/appearance*						
* n*	*33*	*32* ^d^	*33*			
Mean [%]	**50.0**	**89.5**	**92.7**	<0.0001	0.169	0.860
SD	24.8	11.3	9.3			
*Sense*						
* n*	*31*	*32* ^d^	*33*			
Mean [%]	**46.8**	**77.6**	**83.4**	0.0001	0.209	0.696
SD	33.1	23.6	19.4			
*Speech*						
* n*	*33*	*32* ^d^	*33*			
Mean [%]	**68.2**	**89.5**	**92.1**	0.0007	0.203	0.160
SD	26.7	12.6	9.2			
*Self-esteem*						
* n*	*33*	*32* ^d^	*33*			
Mean [%]	**65.2**	**89.4**	**90.7**	<0.0001	0.486	0.306
SD	26.5	11.0	11.9			

^a^Paired  *t*-test for changes between P and D. ^b^Linear mixed models to calculate the further progression from D to 1 y (D and 1 y in relation to the baseline value P). ^c^Linear mixed models to evaluate the effect of the implant type (Ace/Max). ^d^One file is missing for D.
